# Early CT scanning in the emergency department in patients with penetrating injuries: does it affect outcome?

**DOI:** 10.1007/s00068-017-0831-5

**Published:** 2017-09-04

**Authors:** W. J. van den Hout, G. M. van der Wilden, F. Boot, F. J. Idenburg, S. J. Rhemrev, R. Hoencamp

**Affiliations:** 1 Leiden University Medical Center, Albinusdreef 2, 2333 ZA Leiden, The Netherlands; 2 Department of Surgery, Haaglanden Medical Center, Lijnbaan 32, 2512 VA The Hague, The Netherlands; 3 Division of Surgery, Department of Traumatology, Alrijne Hospital, Simon, Smitweg 1, 2353 GA Leiderdorp, The Netherlands; 4 Ministry of Defense, The Hague, The Netherlands

**Keywords:** Penetrating injury, Diagnostics, Trauma, CT scan

## Abstract

**Background:**

To be a level I trauma center in the Netherlands a computed tomography (CT) scanner in the emergency department (ED) is considered desirable, as it is presumed that this optimizes the diagnostic process and that therapy can be directed based on these findings. Aim of this study was to assess the effects of implementing a CT scanner in the ED on outcomes in patients with penetrating injuries.

**Methods:**

In this retrospective descriptive study, patients with penetrating injuries (shot and/or stab wounds), presented between 2000 and 2014 were analysed using the hospital’s electronic database, and data from the West Netherlands trauma registry and the financial department.

**Results:**

405 patients were included: performing a CT scan upon arrival increased significantly from 26.7 to 67.0% (*p* = 0.00) after implementation of a CT scanner in the ED, with the mean cost of a CT being 96.85 euros. Overall mortality decreased from 6.9 to 3.7%, although not statistically significant. Intensive care unit admission (ICU-admission) and median hospital length of stay (H-LOS) decreased from 30.9 to 24.5% resp. 3.2 to 1.8 days (*p* ≤ 0.05). Overall mortality, adjusted for injury severity score (ISS), revised trauma score (RTS), and types of injuries, did not change significantly.

**Conclusion:**

Patients with penetrating injuries more often received a CT scan on admission after implementation of a CT scanner in the ED. Early CT scanning is useful since it significantly reduces ICU-admissions and decreases H-LOS. It is a cheap and non-invasive diagnostic tool with significant clinical impact, resulting in directed treatment, and improvement of outcomes.

## Introduction

Trauma systems were introduced almost 40 years ago. Proof of their effectiveness is an ongoing issue [1] with many studies on the outcomes written in the past decades [2–5]. During the 1990s the Dutch trauma society, collaborating with all other trauma care partners, initiated several projects on improving quality of care for polytrauma patients [6], ranging from the introduction of modern programmes (such as Advanced Trauma Life Support) for teaching and training, to regionalization of specialized trauma care. As a result, trauma centers with three levels of care, with different criteria were designated in the Netherlands. The first Dutch level I trauma center was initiated in 1999 and since then eleven trauma centers were designated as a level I. To be categorized as a level I, immediate availability of ultrasonography (FAST), computed tomography (CT) scanner, angiography, intensive care beds, a stand-by operating room, and surgical team in the ED are obligatory [7].

In the Netherlands, several studies have been published evaluating the effect of the implementation of designated centers; these data show a reduction in overall mortality [8–10]. So far, none of these studies have specifically assessed the impact of the direct availability of a CT scanner. Medical Center Haaglanden (MCH) part of the trauma center West Netherlands (TCWN), with 50,000 patients per year presenting at the ED (including 0.2% patients with penetrating injuries) implemented a CT scanner [General Electric (GE) bright speed 16 slides] in the ED in 2007. Early CT scanning can detect (potentially life threatening) injuries in an early stage, since it is easily accessible, reduces time to diagnosis and there are less patient transfers to a location elsewhere in the hospital [11, 12]. Furthermore, several other studies analysed whether performing a CT scan results in accurate decision making in patients with penetrating injury [13–19].

The aim of this study was to analyse the effects of implementing a CT scanner in the ED in a level I trauma center in the Western part of the Netherlands, for patients with penetrating injuries. We hypothesized that a CT scanner in the ED would result in an increased usage and as a consequence improved outcomes in patients with penetrating injuries.

## Methods

### Data

This is a retrospective cohort study conducted at MCH. Patients were included from January 2000 until December 2014.

Inclusion criteria were hostile penetrating injuries (stab, shot wound or both) and auto mutilation, during the period 2000–2006 (before implementation of the CT scanner) or 2008–2014 (after implementation of the CT scanner), with a subsequent admission after assessment at the ED.

Exclusion criteria were immediate discharge after presenting at the ED, common domestic injuries, lacking data, and patients treated in the year 2007. The last group was excluded, since this was the year of implementation of the CT scanner in the ED, and therefore it was marked as a transition period.

The electronic database was comprised of data collected from the electronic patient files of MCH. Variables as Revised Trauma Score (RTS) [20], Injury Severity Score (ISS) [21], and systolic blood pressure (SBP) were collected from the trauma registry West Netherlands.

The collected variables for each patient included: demographic data (age, gender, type of injury, the localisation, the number of wounds), vital signs (hemodynamic instability and respiratory insufficiency, SBP), RTS, ISS, imaging diagnostics, interventions, hospital length of stay (H-LOS) in days, intensive care unit (ICU) admission, ICU length of stay (ICU-LOS) in days, and mortality.

Types of injuries were divided into shot wounds, stab wounds, or both. The only patient with both shot and stab wounds was categorized as shot wounds, those being the main reason for hospitalisation. The localisation of injuries was divided into head/neck, abdomen, chest, and extremity. The number of wounds was dichotomized into single or multiple wounds.

### Financial data

For the calculation of the costs, data were used from the financial department of the hospital. All declared costs of a CT scan were summed up and divided through the number of CT scans found in the financial data, resulting in the mean cost of a single CT scan.

### Definitions

Patients were scored as being hemodynamic unstable when the heart rate increased ≥120 beats per minute and the blood pressure decreased ≤90 mmHg (signs of significant blood loss). In case no vital signs were documented, hemodynamic instability was also scored when the patient received fresh froze plasma (FFP) or packed red blood cells (PRBC), or when other clinical signs of hemodynamic instability were specifically reported [22]. Patients were scored as being respiratory insufficient when they were cardiopulmonary resuscitated or intubated, or when showing signs of respiratory insufficiency (pO2 <90% or obstruction of the airway). Patients were defined as (too) unstable when they were respiratory insufficient or hemodynamic unstable.

Used imaging diagnostics were chest X-ray, extremity X-ray, FAST, CT scan, and other. The other diagnostics included: other types of X-rays than mentioned above, magnetic resonance imaging, gastroduodenoscopy, bronchoscopy, angiography, and embolization.

The interventions included laparotomy, thoracotomy, and other. The other interventions included: clinical wound excision and wound debridement, bullet removal, stabilizing fractures with osteosynthesis, tracheostoma, fasciotomy, suturing tendon and nerve injury, craniotomy, cerebral ventriculography, intubation and resuscitation.

### Statistical analysis

Data were analysed using SPSS (version 20, IBM Corporation, Armonk, New York). The before (2000–2006) and after group (2008–2014) were compared. The independent *t* test and the Mann–Whitney *U* test were used to compare continuous data and the *χ* ^2^-test to compare the categorical data. Continuous variables were summarized using mean values with standard deviations (SD) or median with interquartile, and categorical variables were reported as counts and proportions. The odds ratio (OR), hazard ratio (HR) and the 95% confidence intervals were calculated to find out whether implementation of the CT scanner resulted in differences in mortality, number of interventions, number of imaging diagnostics, H-LOS, ICU-admission, and ICU-LOS. Logistic regression was used for categorical data and cox regression was used for continuous data. The outcome measures were adjusted for the effect of confounders. A *p* value ≤0.05 was considered significant. The institutional review board of MCH approved the study.

## Results

**Fig. 1:**
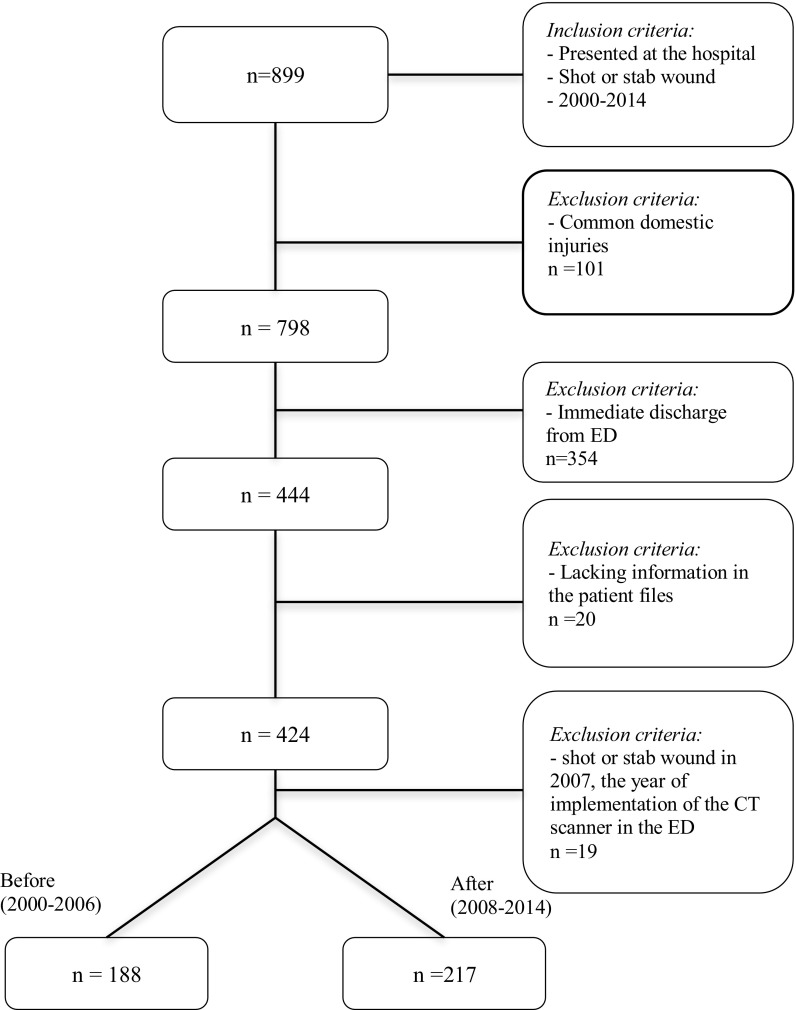
Flow chart, included and excluded patients. *n* number, *ED* emergency department, *CT* computed tomography

A total of 899 patients with penetrating injuries presented at the ED between 2000 and 2014. A total of 575 patients were excluded (Fig. 1), resulting in 424 eligible patients: 217 in the before (2000–2006) and 188 in the after group (2008–2014).

### Comparing the groups before and after implementing the CT scanner

The demographic characteristics were comparable between the two periods (Table 1).

**Table 1:** Patient characteristics

	2000–2006	2008–2014	*p* value^a^
Patients, *n*	217	188	–
Male, *n* (%)	197 (90.8)	172 (91.5)	0.803
Mean age, years ± SD	31.17 ± 11.63	32.46 ± 12.07	0.274
Stab wound, *n* (%)	154 (71.0)	152 (80.9)	0.021
Shot wound, *n* (%)	63 (29.0)	36 (19.1)	0.021
Shot wound to the head/neck, *n* (%)	18 (8.3)	5 (2.7)	0.015
Multiple wounds, *n* (%)	86 (39.6)	87 (46.3)	0.178
Hemodynamic instability, *n* (%)	36 (16.6)	33 (17.6)	0.797
Respiratory insufficient, *n* (%)	30 (13.8)	22 (11.7)	0.524
Patients, *n*	133	188	–
Median SBP, mmhg (i.q.r.)	135.5 (120.0–150.0)	133 (115–150)	0.729
Median RTS (i.q.r.)	7.84 (7.84–7.84)	7.84 (7.77–7.84)	0.003
Median ISS (i.q.r.)	9 (1.0–10.0)	9 (2.0–12.5)	0.665
ISS ≥16, *n* (%)	20 (15.0)	33 (17.6)	0.991

*SBP* systolic blood pressure, *RTS* revised trauma score, *ISS* injury severity score, *n* number, *i.q.r.* inter quartile range

^a^ Student’s *t* test; Chi-square test; Mann–Whitney *U* test

The number of shot wounds decreased while the number of stab wounds increased between the periods (*p* ≤ 0.05).

Additionally, shot wounds to the head decreased from 8.3 to 2.7% (*p* ≤ 0.05).

Data from the trauma registry were only available from 2003 onwards, so the eligible number of patients in the first period decreased to 133 instead of 217 patients for the variables SBP, RTS and ISS.

20.0% (4/20) of the patients who have scored an ISS ≥16 before 2007 died vs. 21.2% (7/33) after 2007, (*p* > 0.05) and 60.0% (12/20) vs. 72.7% (24/33) received a CT scan (*p* > 0.05).

In the group before 2007, 13.4% (29/217) and in the group after 2007, 7.4% (14/188) patients were too instable to receive a CT scan (*p* = 0.054). In this subgroup, mortality was 34.5% (10/29) and 35.7% (5/14) before and after 2007(*p* > 0.05).

The anatomic distribution of the penetrating injuries before and after 2007 did not change: extremities 35.5 vs. 44.1%, chest 43.8 vs. 44.7%, abdomen 36.4 vs. 33.0% and head/neck 20.3 vs. 23.4% (*p* > 0.05).

Using the CT scanner at arrival as a diagnostic tool increased significantly from 26.7% (58/217) to 67.0% (126/188), *p* = 0.00. Details of other diagnostic procedures, interventions performed, and main outcomes are shown in Table 2. The four largest groups within other interventions included intubation and resuscitation (26.1%), suturing tendon and nerve injury (19.8%), clinical wound excision or wound debridement (13.5%), and stabilizing fractures with osteosynthesis (9.9%).

**Table 2:** Patient outcomes before and after 2007

	2000–2006	2008–2014	*p* value^a^
Patients, *n*	217	188	–
CT, *n* (%)	58 (26.7)	126 (67.0)	0.000*
Chest X-ray, *n* (%)	167 (77.0)	151 (80.3)	0.411
FAST, *n* (%)	119 (54.8)	95 (50.5)	0.387
Extremity X-ray, *n* (%)	41 (18.9)	31 (16.5)	0.528
Other diagnostics, *n* (%)	59 (27.2)	43 (22.9)	0.318
Thoracotomy, *n* (%)	9 (4.1)	9 (4.8)	0.755
Laparotomy, *n* (%)	43 (19.8)	28 (14.9)	0.194
Other interventions, *n* (%)	61 (28.1)	50 (26.6)	0.733
Mortality, *n* (%)	15 (6.9)	7 (3.7)	0.158
Median H-LOS, days (i.q.r.)	3.208 (1.242–6.879)	1.799 (0.772–4.494)	0.002*
ICU-admission, *n* (%)	67 (30.9)	46 (24.5)	0.018*
Median ICU-LOS, days (i.q.r.)	0.811 (0.513–1.984)	0.997 (0.581–2.539)	0.201

*CT* computed tomography, *FAST* ultrasonography, *H-LOS* hospital length of stay, *ICU* intensive care unit, *ICU-LOS* intensive care unit length of stay, *n* number, *i.q.r.* inter quartile range

^a^ Chi-square test; Mann–Whitney *U* test

The overall mortality decreased from 6.9 to 3.7%, although not statistically significant (*p* = 0.158). Number of ICU-admission and median H-LOS decreased from 30.9 to 24.5%, respectively, 3.2 to 1.8 days, both being significant at *p* ≤ 0.05.

Table 3 shows the analysis of the effect on using the CT scanner on clinical outcomes (such as mortality, H-LOS, ICU-admission, and ICU-LOS), adjusted for type of injury, ISS, and RTS.

**Table 3:** Outcomes after implementation of the CT scanner in the ED adjusted for ISS, RTS, and type of injury

	Test	Odds ratio/hazard ratio (95% CI)	*p* value^a^
CT usage	Log regression	5.616 (3.276–9.626)	0.000*
Mortality	Log regression	2.950 (0.221–39.427)	0.413
H-LOS	Cox regression	1.183 (0.926–1.512)	0.178
ICU-admission	Log regression	0.639 (0.337–1.209)	0.169
ICU-LOS	Cox regression	0.627 (0.391–1.006)	0.053

*Log* binary logistic, *Cox* proportional hazard, *CT* computed tomography, *H-LOS* hospital length of stay, *ICU* intensive care unit, *ICU-LOS* intensive care unit length of stay, *RTS* revised trauma score, *ISS* injury severity score

^a^Adjusted for ISS, RTS, type of injury

The usage of chest X-ray, FAST, extremity X-ray and other diagnostics adjusted for the RTS, ISS and type of injury did not change (*p* > 0.05). Additionally, no significant differences were found in the number of interventions performed: laparotomy, thoracotomy and other interventions adjusted for ISS, RTS and type of injury (*p* > 0.05).

The overall costs of performing a CT scan (in both periods together) were found to be 34,577.19 euros. In total 357 CT scans were made, resulting in an average price of 96.85 euro per CT scan.

## Discussion

This study analysed 14 years of data, including more than 400 patients with penetrating trauma within the region of MCH, The Hague, in the Netherlands. The implementation of a CT scanner in the ED in 2007 contributed to an increase of its use. The median H-LOS and number of ICU-admissions decreased significantly (*p* ≤ 0.05). Additionally, there was a decreasing tendency in performing interventions, in particularly laparotomies (19.8–14.9%), while there was also a trend towards a decreased overall mortality (6.9–3.7%).

During the 1990’s many studies were published on the effect of implementing a level I trauma center [2–5]. To be a level 1 trauma center a CT scanner in the ED is considered desirable [7]. Other studies analysed other aspects of having the CT scanner in the ED: time till diagnosis, time from first arrival in the ED to first CT imaging, and overall time spent in the ED [23–25]. However, of those studies only Saltzherr et al. and Lee et al. focussed on clinical outcomes, both showing no significant differences in 30-day and 1-year mortality, H-LOS, and ICU-LOS. They concluded that implementing a CT scanner in the ED showed a faster availability of first CT [11, 26]. However, the power of the study of Saltzherr et al., using prespecified groups (multiple trauma patients and severe trauma brain injury patients), was low [11].

Analysing the impact on outcomes after implementing a CT scanner in the ED, we specifically studied patients with shot or stab wounds. We found a significant increase in the use of the CT scanner, which could be explained through logistics (the scanner being so close by). The current literature confirms the trend of the use of more defensive medical care, which may explain the increase in the use of CT scan in our study [27]. This increase in use often results in higher costs [27, 28]. Additionally, a CT scan has become the ‘gold standard’ as definitive diagnostic imaging of most injuries in severely and potentially severely injured patients. However, too severely injured patients will receive emergency surgery without diagnostic procedures, because of time pressure. Having the CT scanner in the ED improves workflow, since it reduces time from arrival to first CT, because it reduces the time of patient transfer to a CT scanner elsewhere in the hospital resulting in early diagnosis and goal-directed therapy [11, 12].

Minor downside of performing more CT scans is the increased radiation exposure per patient [29]. Mean effective dose of a CT scan abdomen compared to an X-ray (exclusive chest X-ray) for each intervention was 10.28 millisievert (mSv) against 0.13 mSv (measured in the Netherlands in 2013), although there is a wide range of dose exposure per intervention per body area. Abdominal CT scanning contributes most to radiation exposure of all CT scans [30, 31]. However, developments in technology as well as modified protocols will result in a decreased radiation exposure [32].

Our study shows a decreasing trend in overall mortality from 6.9 to 3.7%. Performing a CT scan more often and earlier reduces time till diagnosis and facilitates faster decision making for therapeutic interventions [24, 25], which could result in a decreasing overall mortality. Additionally, the significant decrease in shot wounds and shot wounds to the head after 2007 could also contribute to a decreasing overall mortality, considering that the outcomes after shot wounds, especially to the head is worse than for stab wounds in general.

Other studies have found that performing CT scan in patients with penetrating injuries in general decreases mortality [33, 34], which is in line with our study. However, our results are not statistically significant, which can be explained by the fact that our sample size is too small. The trend towards a decreasing overall mortality rate after implementation of a level I trauma center was confirmed by other Dutch studies [8–10].

Another focus point was the effect of CT scanning on performing interventions; our study shows a decrease in patients undergoing exploratory laparotomy and other interventions. It also showed a decrease in H-LOS and number of ICU-admissions, which is in line with current literature. Grossman et al. concluded that performing a CT scan in patients with penetrating injuries results in more accurate decision making in performing other imaging diagnostics [14]. Performing a CT scan can also safely limit the use of surgical interventions (reducing negative exploratory laparotomies) or predict the need for it [13, 16, 18, 19], resulting in earlier discharge of patients after a shorter period of observation [35, 36]. The CT scan is a diagnostic tool with a high sensitivity, specificity and accuracy, to detect peritoneal violation and predict the need for a laparotomy in patients with penetrating injuries [13, 37, 38]. In the review of Goodman et al., pooled weighted estimates of sensitivity specificity and accuracy of the CT scan to predict the need for laparotomy were 94.9, 95.38 and 94.7%, respectively [39]. However, bowel injuries are challenging to diagnose on CT, using a variety of CT criteria can achieve accurate results [40].

Another variable is the costs of these additional CT scans, especially when plotted against the clinical relevance they have.

Based on the costs calculated in the REACT-trial [41] (a hospital general ward day costing 504 euros and an ICU day 1782 euros), performing a CT scan seems to be a non-expensive intervention, less than 100 euro per CT scan. So while the number of CT scans performed increases, resulting in higher overall costs for diagnostic imaging, it results in a decrease in H-LOS, number of ICU-admissions, and number of laparotomies, making it a cost-effective tool. The REACT-trial also confirmed a decrease in costs of surgical interventions in hospitals with early CT in the ED compared to a hospital without direct availability of a CT scanner in the ED.

Our study has several limitations, primarily since it is retrospective in nature. The data used from the trauma registry were only available from 2003 onwards. Data were sometimes incomplete.

It was not possible to adjust for all possible confounders such as improvements in medical care, medical knowledge, technical skills, imaging techniques, and hospital care, which all could have contributed to better care and subsequently influence our outcomes. Furthermore, selective non-operative management can be safely applied in patients with penetrating injuries [42–44]. During the 14-year period, patients were possibly treated more often by principles of selective non-operative management, which could subsequently influence our outcomes as well.

Our study does not include the logistic part behind implementation of a CT scanner in the ED (variables such as time till diagnosis, mean time duration from arrival to first CT, and time in the ED). In the REACT-trial mean time from arrival till first CT after implementation of a CT scanner in the ED is reduced by 13 min (median time 49 min vs. median time of 36). The hospital in the REACT-trail and our hospital are both level I trauma centers with a CT scanner in the ED in the Netherlands. Therefore, the mean time reduction from arrival till first CT will also be around 13 min in our hospital after implementation of the CT scanner in the ED [11].

We recommend a larger prospective study in the future to look at the effect of the CT scanner in the ED in patients with penetrating injuries, focusing on clinical outcomes, time-aspects and provide a detailed cost-effective analysis.

In conclusion, the availability of a CT scanner in the ED especially in patients with penetrating injuries, results in more goal-directed treatment, and improvement of outcomes; it significantly reduces ICU-admissions and decreases H-LOS. Therefore, we can and should use it as a relatively cheap and non-invasive diagnostic tool due to its significant clinical impact.
